# Therapeutic Effect of *Dipsacus asperoides* C. Y. Cheng et T. M. Ai in Ovalbumin-Induced Murine Model of Asthma

**DOI:** 10.3390/ijms20081855

**Published:** 2019-04-15

**Authors:** Na-Rae Shin, A Yeong Lee, Gunhyuk Park, Je-Won Ko, Jong-Choon Kim, In-Sik Shin, Joong-Sun Kim

**Affiliations:** 1 College of Veterinary Medicine (BK21 Plus Project Team), Chonnam National University, 77 Yongbong-ro, Buk-gu, Gwangju 61186, Korea; tlsskfo870220@gmail.com (N.-R.S.); rheoda@gmail.com (J.-W.K.); toxkim@jnu.ac.kr (J.-C.K.); 2 Herbal Medicine Resources Research Center, Korea Institute of Oriental Medicine, Geonjae-ro 177, Naju-si, Jeollanam-do 58245, Korea; lay7709@kiom.re.kr (A.Y.L.); gpark@kiom.re.kr (G.P.)

**Keywords:** *Dipsacus asperoides* C. Y. Cheng et T. M. Ai, allergic asthma, pro-inflammatory cytokine, inducible nitric oxide synthase, nuclear factor kappa B

## Abstract

*Dipsacus asperoides* C. Y. Cheng et T. M. Ai (DA) has been used in China as a traditional medicine to treat lumbar and knee pain, liver dysfunction, and fractures. We explored the suppressive effect of DA on allergic asthma using an ovalbumin (OVA)-induced asthma model. In the asthma model, female Balb/c mice were sensitized to OVA on day 0 and 14 to boost immune responses and then exposed to OVA solution by using an ultrasonic nebulizer on days 21 to 23. DA (20 and 40 mg/kg) was administered to mice by oral gavage on days 18 to 23. Methacholine responsiveness was determined on day 24 using a plethysmography. On day 25, we collected bronchoalveolar lavage fluid, serum, and lung tissue from animals under anesthesia. DA treatment effectively inhibited methacholine responsiveness, inflammatory cell infiltration, proinflammatory cytokines such as interleukin (IL)-5 and IL-13, and immunoglobulin (Ig) E in OVA-induced asthma model. Reductions in airway inflammation and mucus hypersecretion, accompanied by decreases in the expression of inducible nitric oxide synthase (iNOS) and the phosphorylation of nuclear factor kappa B (NF-κB), were also observed. Our results indicated that DA attenuated the asthmatic response, and that this attenuation was closely linked to NF-κB suppression. Thus, this study suggests that DA is a potential therapeutic for allergic asthma.

## 1. Introduction

Allergic asthma is a chronic inflammatory respiratory disease mediated by general environmental allergens and accompanied by symptoms such as wheezing, coughing, and airway remodeling [1]. The disease is featured as eosinophilic airway inflammation, mucus secretion, and airway hyperresponsiveness [2], and the development of the disease involves various cells, such as T-helper type 2 (Th2) cells, mast cells, and eosinophils [3]. In particular, the elevation in pro-inflammatory cytokines, including interleukin (IL)-4, IL-5, and IL-13, and the number of eosinophils are considered important factors in the pathogenesis of allergic asthma [4]. The production of pro-inflammatory cytokines induces immunoglobulin E (IgE) switching in B cells, the recruitment of eosinophils, and the production of various inflammatory mediators [5,6]. Therefore, many researchers have conducted experiments that explore the reduction in the release of pro-inflammatory cytokines as a strategy for controlling allergic asthma [7].

In allergic asthma, nitric oxide (NO) plays an important role in airway inflammation [8]. NO has three isoforms: neuronal NOS (nNOS), endothelial NOS (eNOS), and inducible NOS (iNOS) [9]. iNOS is particularly involved in the elevation of inflammatory cell infiltration into asthmatic lesions, which is stimulated by many pro-inflammatory cytokines [8]. The increased expression of iNOS induces the elevation of NO production in patients with asthma and activates T-helper 2 lymphocytes [10,11]. These events eventually induce airway inflammatory responses via the elevation of inflammatory mediators. In addition, NF-κB is a transcription factor and is considered as an important factor in the pathogenesis of asthma because its activation induces inflammatory and immune responses [12,13]. The activation of NF-κB, which is increased in the airways of patients with asthma and in asthmatic animals, induces the production of pro-inflammatory mediators. In contrast, a reduction in the activation of NF-κB mitigates the clinical signs of asthma [14,15]. Thus, the downregulation of NF-κB may be a target for the treatment of allergic asthma.

Dipsaci Radix, a dry root of Dipsacus asperoides C. Y. Cheng et T. M. Ai (DA), is distributed widely in the southwestern part of China and has been used as a traditional medicine to treat lumbar and knee pain, liver dysfunction, traumatic hematoma, threatened abortion, and bone fractures [16,17,18,19,20]. Based on the ethnopharmacological evidence of DA, many researchers have investigated its protective effect against bone loss [21,22]. Recent studies have shown other properties of DA such as antioxidant and anti-inflammatory effects [23,24,25,26]. DA has protective effects against collagen-induced arthritis in mice amd LPS-induced inflammation in RAW 264.7 mouse macrophages [23,26]. Particularly, saponin, the active ingredient of DA, displayed anti-inflammatory properties in various experiments [20,27,28,29,30]. Considering these properties of DA, we hypothesized that DA has a protective effect on allergic asthma. However, there has been no study on the pharmacological properties of DA against allergic inflammation until now.

The goal of our study is to evaluate whether DA is effective against allergic asthma induced by OVA exposure. To evaluate the anti-inflammatory effect of DA, we measured AHR, inflammatory cell counts, cytokines, and the expression of iNOS and NF-κB, in a mouse model of OVA-induced allergic asthma.

## 2. Results

### 2.1. HPLC Analysis of DA

**Figure 1:**
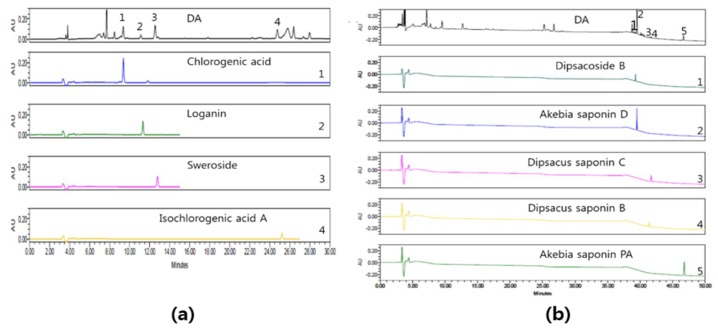
Chromatogram of *Dipsacus asperoides* C. Y. Cheng et T. M. Ai (DA) detected at 237 (**a**) and 198 nm (**b**).

HPLC chromatogram of DA is presented in Figure 1. The nine compounds found were chlorogenic acid, loganin, sweroside, isochlorogenic acid, dipsacoside B, akebia saponin D, dipsacus saponin C, dipsacus saponin B, and akebia saponin PA; these compounds were found at approximately 9.4, 11.3, 12.8, 25.2, 39.2, 39.4, 41.4, 41.6, and 44.8 min, respectively. Four components of DA (chlorogenic acid, loganin, sweroside, and isochlorogenic acid) were detected at 237 nm (Figure 1a), and five saponins (dipsacoside B, akebia saponin D, dipsacus saponin C, dipsacus saponin B, and akebia saponin PA) were detected at 198 nm (Figure 1b), respectively. The chlorogenic acid, loganin, sweroside, isochlorogenic acid A, dipsacoside B, akebia saponin D, dipsacus saponin C, dipsacus saponin B, and akebia saponin PA contents were 3.20 ± 0.018, 1.51 ± 0.016, 7.16 ± 0.142, 3.65 ± 0.121, 8.56 ± 0.485, 166.33 ± 1.500, 4.13 ± 0.024, 2.78 ± 0.043, and 11.10 ± 0.081 μg/mg, respectively. Of these nine components, akebia saponin D was detected as the major component.

### 2.2. DA Reduces Methacholine Responsiveness in OVA-Induced Asthma Model

**Figure 2:**
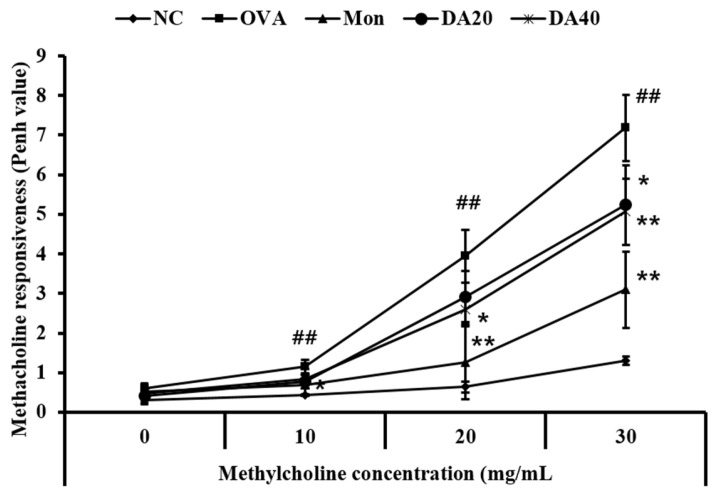
DA decreased methacholine responsiveness in ovalbumin (OVA)-induced asthma model. DA-treated mice exhibited a significant reduction in methacholine responsiveness after OVA exposure. NC: normal controls, PBS treatment, and inhalation; OVA: asthma model, PBS treatment, and OVA sensitization and inhalation; Mon: montelukast-treated mice, montelukast (10 mg/kg, oral gavage), and OVA sensitization and inhalation; DA20 and 40: *Dipsacus asperoides* C. Y. Cheng et T. M. Ai-treated mice (20 and 40 mg/kg, respectively, oral gavage) and OVA sensitization and inhalation. The values shown are the mean ± SD. ## *p* < 0.01 *vs.* NC; * *p* < 0.05, ** *p* < 0.01, respectively.

The methacholine responsiveness of OVA-induced asthma model increased in comparison with those of normal controls, with an increase in methylcholine concentration (Figure 2). However, montelukast-treated mice exhibited significantly lower methacholine responsiveness than those of OVA-induced asthma model. DA-treated mice showed a reduction in methacholine responsiveness compared with OVA-induced asthma model, with an increase in methylcholine concentration. These reductions were observed in the 40 mg/kg group.

### 2.3. DA Suppress the Inflammatory Cell Counts in BALF from OVA-Induced Asthma Model 

**Figure 3:**
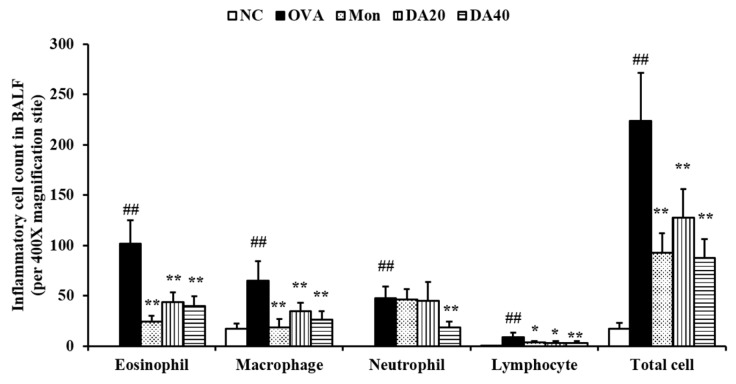
DA decreased the number of inflammatory cells in bronchoalveolar lavage fluid (BALF) of OVA-induced asthma model. DA-treated mice exhibited a marked decline in inflammatory cell counts compared with OVA-induced asthma model. NC: normal controls, PBS treatment and inhalation; OVA: PBS treatment with OVA sensitization and inhalation; Mon: montelukast-treated mice (10 mg/kg, oral gavage) with OVA sensitization and inhalation; DA20 and 40: Dipsacus asperoides C. Y. Cheng et T. M. Ai-treated mice (20 and 40 mg/kg, respectively, oral gavage) with OVA sensitization and inhalation. The values shown are the mean ± SD. ## *p* < 0.01 *vs.* NC; * *p* < 0.05, ** *p* < 0.01, respectively.

**Figure 4:**
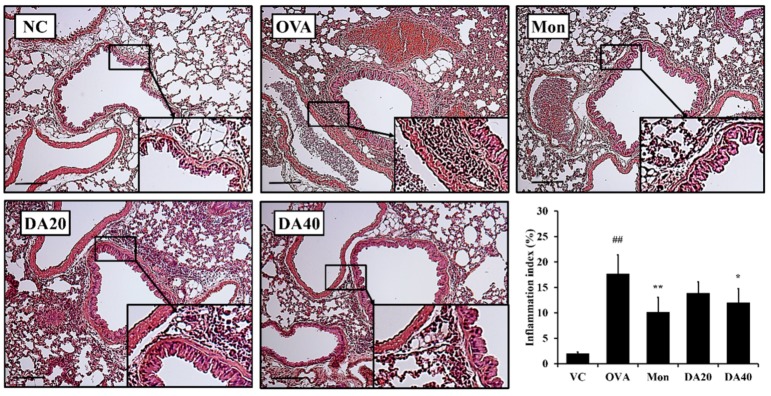
DA suppressed inflammatory cell infiltration in the lung tissue of OVA-induced asthma model. The DA-treated mice exhibited lower inflammatory cell infiltration into the lung tissue than OVA-induced asthma model. NC: normal controls, PBS treatment and inhalation; OVA: PBS treatment with OVA sensitization and inhalation; Mon: montelukast-treated mice (10 mg/kg, oral gavage) with OVA sensitization and inhalation; DA20 and 40: *Dipsacus asperoides* C. Y. Cheng et T. M. Ai-treated mice (20 and 40 mg/kg, respectively, oral gavage) with OVA sensitization and inhalation. Scale bar = 50 µm. The values shown are the mean ± SD. ## *p* < 0.01 *vs.* NC; * *p* < 0.05, ** *p* < 0.01, respectively.

The OVA-induced asthma model exhibited a marked increase in the number of inflammatory cells, particularly eosinophils, compared with the normal control mice (Figure 3). However, montelukast-treated mice exhibited significantly lower inflammatory cell counts than those of OVA-induced asthma model. Similar to results from montelukast-treated mice, DA-treated mice exhibited significantly lower inflammatory cell counts than those of OVA-induced asthma model. These results were consistent with the results of the histological analysis (Figure 4). The OVA-induced asthma model showed greater infiltration of inflammatory cells around the bronchi than in the normal control mice group. In contrast, in DA-treated mice, the infiltration of inflammatory cells decreased compared to that in OVA-induced asthma model.

### 2.4. DA Reduced The Levels of IL-5, IL-13, Eotaxin, and Total Ig E in OVA-Induced Asthma Model

**Figure 5:**
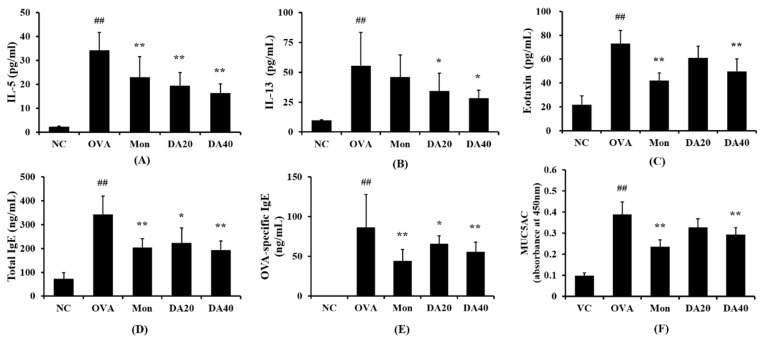
DA decreased the level of Th2 cytokines and IgE in OVA-induced asthma model. (**A**) IL-5, (**B**) IL-13, (**C**) Eotaxin, (**D**) Total IgE, (**E**) OVA-specific IgE, and (**F**) MUC5AC. NC: normal controls, PBS and inhalation; OVA: PBS treatment with OVA sensitization and inhalation; Mon: montelukast-treated mice (10 mg/kg, oral gavage) with OVA sensitization and inhalation; DA20 and 40: *Dipsacus asperoides* C. Y. Cheng et T. M. Ai-treated mice (20 and 40 mg/kg, respectively, oral gavage) with OVA sensitization and inhalation. The values shown as the mean ± SD. ## *p* < 0.01 *vs.* NC; * *p* < 0.05, ** *p* < 0.01, respectively.

The level of IL-5 in BALF was significantly higher in OVA-induced asthma model group than in the non-induced group (Figure 5A). In contrast, montelukast-treated mice had significantly lower levels of IL-5 in BALF than OVA-induced asthma model, and DA-treated mice had markedly lower levels of IL-5 than OVA-induced asthma model. Similarly, the levels of IL-13 and eotaxin were also higher in OVA-induced asthma model than in the normal mice (Figure 5B,C, respectively), whereas DA-treated mice had significantly lower levels than OVA-induced asthma model.

The level of total and OVA-specific IgE in serum was higher in OVA-induced asthma model than in the normal control mice (Figure 5D,E, respectively). In contrast, DA-treated mice exhibited a significant reduction in total and OVA-specific IgE in serum compared with OVA-induced asthma model.

### 2.5. DA Decreased Expression of iNOS and Phosphorylation of NF-κB in OVA-Induced Asthma Model

**Figure 6:**
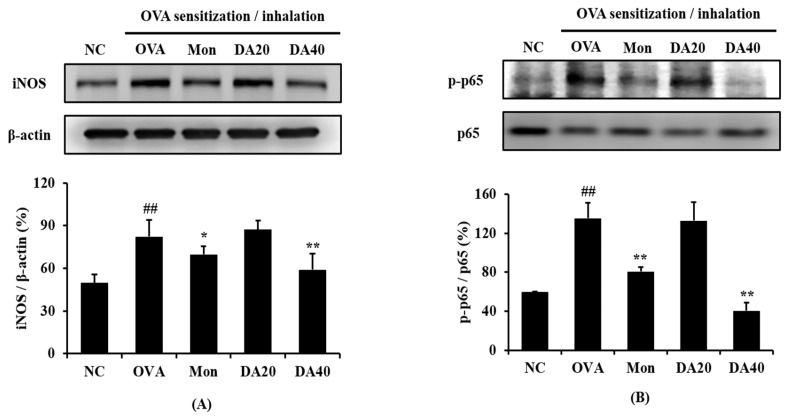
DA inhibited the expression of iNOS and the phosphorylation of NF-κB in OVA-induced asthma model. Densitometric band value was determined using ChemiDoc (Bio-Rad Laboratories, Hercules, CA, USA). Relative expression value is expressed as iNOS *vs.* β-actin and p-p65 vs p65. (**A**) Expression of iNOS; (**B**) expression of phosphorylation of NF-κB. Relative expression value NC: normal controls, PBS treatment and inhalation; OVA: PBS treatment with OVA sensitization and inhalation; Mon: montelukast-treated mice (10 mg/kg, oral gavage) with OVA sensitization and inhalation; DA20 and 40: *Dipsacus asperoides* C. Y. Cheng et T. M. Ai-treated mice (20 and 40 mg/kg, respectively, oral gavage) with OVA sensitization and inhalation. The values shown are the means ± SD. ## *p* < 0.01 *vs.* NC; * *p* < 0.05, ** *p* < 0.01, respectively.

**Figure 7:**
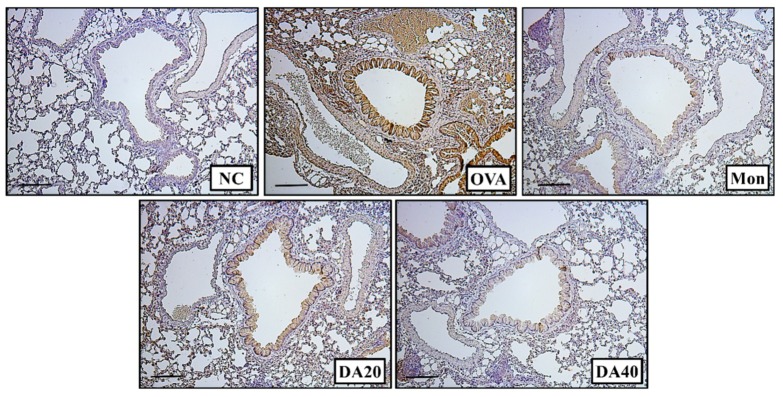
DA decreased iNOS expression in the lung tissue of OVA-induced asthma model. DA-treated mice exhibited a reduction in iNOS expression in the lung tissue compared with OVA-induced asthma model. NC: normal controls, PBS treatment and inhalation; OVA: PBS treatment with OVA sensitization and inhalation; Mon: montelukast-treated mice (10 mg/kg, oral gavage) with OVA sensitization and inhalation; DA20 and 40: *Dipsacus asperoides* C. Y. Cheng et T. M. Ai-treated mice (20 and 40 mg/kg, respectively, oral gavage) with OVA sensitization and inhalation. Scale bar = 50 µm.

The expression of iNOS was significantly higher in OVA-induced asthma model than in the normal control mice (Figure 6A), whereas DA-treated mice exhibited a marked decline in iNOS expression compared with OVA-induced asthma model. In addition, phosphorylation of NF-κB in lung significantly increased in OVA-induced asthma model compared to that in normal controls (Figure 6B). However, DA-treated mice exhibited a marked reduction in the phosphorylation of NF-κB compared with OVA-induced asthma model. Immunohistological analysis of the lung tissue revealed that DA treatment effectively decreased iNOS expression in OVA-induced asthma model (Figure 7).

### 2.6. DA-Reduced Mucus Production in OVA-Induced Asthma Model

**Figure 8:**
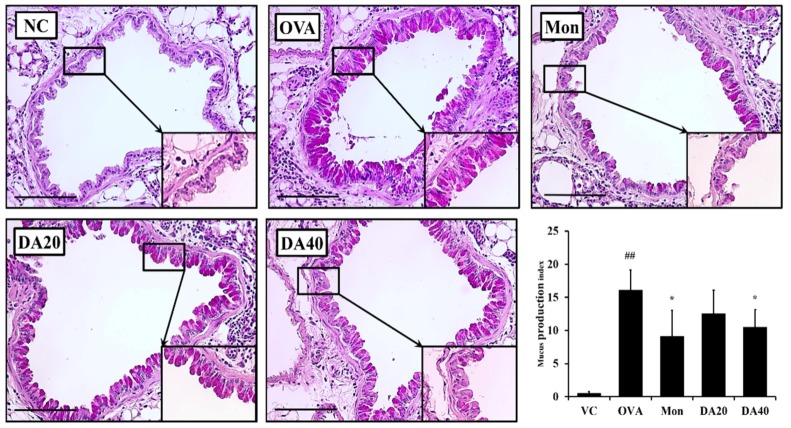
DA reduced mucus production in the lung tissue of OVA-induced asthma model. Lung tissue was stained with PAS solution. NC: normal controls, PBS treatment and inhalation; OVA: PBS treatment with OVA sensitization and inhalation; Mon: montelukast-treated mice (10 mg/kg, oral gavage) with OVA sensitization and inhalation; DA20 and 40: *Dipsacus asperoides* C. Y. Cheng et T. M. Ai-treated mice (20 and 40 mg/kg, respectively, oral gavage) with OVA sensitization and inhalation. Scale bar = 50 µm. The values shown as the mean ± SD. ## *p* < 0.01 vs. NC; * *p* < 0.05.

Mucus production was markedly increased in OVA-induced asthma model compared to that in normal controls (Figure 8). However, montelukast- and DA-treated mice had milder mucus production in the bronchial airway compared with OVA-induced asthma model.

## 3. Discussion

In this study, we explored the effects of DA against OVA-induced allergic asthma and investigated its possible mechanism. DA-treated mice exhibited a marked reduction in methacholine responsiveness, inflammatory cell counts, pro-inflammatory cytokines, and OVA-specific IgE in comparison to those of OVA-induced asthma model. These therapeutic changes were accompanied by histological alterations, including reductions in the airway inflammatory responses and mucus secretion. In addition, DA treatment effectively suppressed the iNOS expression and NF-κB phosphorylation induced by OVA exposure.

Allergic asthma is one of the most prevalent diseases in the world, and the increase in the incidence of asthma is a notable problem [31]. The pathogenesis of allergic asthma is related to an increase in inflammatory cells and mucus production [32]. The exposure of allergens in asthma induces the release of pro-inflammatory cytokines, such as IL-5 and IL-13, and ultimately induces eosinophilic airway inflammation and increases in inflammatory mediators [6,33]. In our study, DA-treated mice exhibited a significantly lower number of inflammatory cells in BALF than OVA-induced asthma model, and lower levels of IL-5 and IL-13. Also, these results were consistent with histological analysis. DA-treated mice exhibited lower inflammatory cell infiltration and mucus production than OVA-induced asthma model. These results indicated that the anti-asthmatic effects of DA were closely related to the decrease in pro-inflammatory cytokines. Furthermore, Th2 cytokines increase IgE switching in B cells, which enhances inflammation and mucus secretion [6]. In our study, total and OVA-specific IgE was markedly decreased by DA-treatment in OVA-induced asthma model. These results were strongly supportive of the therapeutic effect of DA on OVA-induced asthma.

The increase in iNOS expression plays an important role in the development of allergic asthma [34], as it elevates NO production, induces eosinophil infiltration, and exacerbates airway inflammation [4]. In addition, iNOS expression is associated with the release of pro-inflammatory cytokines and the increase in iNOS expression induces the elevation of pro-inflammatory cytokines, which results in enhanced recruitment of inflammatory cells in asthmatic lesions [35]. These events were associated with phosphorylation of NF-κB [15]. In a previous report, a reduction in NF-κB phosphorylation attenuated the inflammatory responses in allergic asthma through a decrease in iNOS expression and pro-inflammatory cytokines [36]. Particularly, p65, subunit of NF-Κb, is phosphoylated by various stimuli and then translocated into nucleus, which eventually transcript various inflammatory mediator such as iNOS, cytokines, and chemokines. Therefore, suppression of NF-κB is an important strategy in controlling inflammatory responses [37]. In this study, DA treatment resulted in a marked reduction in iNOS expression in OVA-induced asthma model. These effects were consistent with the immunohistochemistry results for iNOS expression on lung tissue. In addition, the phosphorylation of NF-κB was lower in DA-treated mice than in OVA-induced asthma model. These results indicated that the DA may be an effective therapeutic agent to treat allergic asthma.

Currently, many researchers have discovered therapeutic agents based on natural productS that are effective in treating allergic asthma. However, it is difficult to determine which of various therapeutic agents are more effective at treating allergic asthma. The advantage of DA is that it can be easily collected compared to other therapeutic agents, which will result in cost saving in drug development. In addition, according to previous study, DA did not any toxic effect on experimental animals to 1000 mg/kg (male) or 2000 mg/kg (female) in subchronic toxicity study [38]. Therefore, DA was considered to be a therapeutic agent with safety and efficacy in asthma. To develop DA as therapeutic agent for treating asthma, various specialists such as pharmacy, pharmacodynamics, and pharmacokinetics are needed.

In this study, DA showed the protective effects on OVA-induced asthma model. However, our results did not clearly explain the mechanism of action of DA. In order to explain the involvement of the potential mechanism, additional experiments using protein inhibitors, siRNA, etc., may be necessary in further study. In addition, further study is needed to determine the effect of active ingredients of DA on OVA-induced asthma. 

In summary, administration of DA in OVA-induced allergic asthmatic animals exhibited antiasthmatic effects including the reduction in AHR, proinflammatory cytokine, IgE, inflammatory cell infiltration, and mucus secretion. These effects were accompanied with decreases in iNOS expression and NF-κB phosphorylation. Based on our results, DA can be used as a therapeutic agent to protect against allergic asthma.

## 4. Materials and Methods 

### 4.1. Plant Materials and Chemicals

*Dipsacus asperoides* C. Y. Cheng et T. M. Ai (DA) was purchased from Naemome Dah Herbal Medicine (Ulsan Metropolitan, Korea), morphological analysis was performed by Dr. Goya Choi, and genetic analysis was performed by Dr. Byeong Cheol Moon of the Korea Institute of Oriental Medicine. A voucher specimen was deposited in the Korean Herbarium of Standard Herbal Resources (No. 2-17-0059~2-17-0060). DA (608.9 g) was refluxed in 70% ethanol for 2 h. The extraction was filtered, and the solvent was removed under vacuum. The yield of the DA powder was 47.86% (*w*/*w*), and it was stored at 4 °C until it was used.

Chlorogenic acid (≥98%) and loganin (≥98%) were purchased from KOC Biotech Corporation (Daejeon, Korea) and Wako Pure Chemical Corporation (Osaka, Japan), respectively. Sweroside (≥97%), isochlorogenic acid A (≥98%), and dipsacoside B (≥98%) were obtained from ChemFaces Biochemical Co., Ltd. (Wuhan, China). Akebia saponin D (≥95%), akebia saponin PA (≥95%), dipsacus saponin B (≥90%), and dipsacus saponin C (≥90%) were obtained from the National Development Institute of Korean Medicine (Gyeongsangbuk-do, Korea)

### 4.2. High-Performance Liquid Chromatography (HPLC) Analysis

Quantitative analysis of ingredients was performed using high-performance liquid chromatography (HPLC) coupled with a photodiode array detector (PDA). The HPLC system (Waters Corporation, Milford, MA, USA) comprised a separation module (Waters e2695) and 2998 PDA detector. The analytical data were processed by using Empower 3 (Waters Corporation). DA (74.9 mg) was dissolved in 10 mL 70% ethanol and passed through a 0.2 μm syringe filter. The separation of compounds was performed on a Kromasil column (5 μm, 4.6 × 250 mm, AkzoNobel, Bohus, Sweden) with an injection volume of 10 μL and a flow rate of 0.8 mL/min. The mobile phase consisted of 0.05% formic acid in distilled water (A) and acetonitrile (B), and the following linear gradient program was used: 95% A–70% A, 0–30 min; 70% A–60% A, 30–35 min; and 60% A–50% A, 35–50 min. The sampler and column temperatures were 4 °C and 30 °C, respectively. Detection wavelengths between 195 and 400 nm were scanned, and the sample peaks were detected at 237 nm and 198 nm.

### 4.3. Experimental Procedure

Ethical and scientific management procedures for all animal experiments were approved by Institutional Animal Care and Use Committee of Chonnam National University. Six-week-old female BALB/c mice were purchased from Samtako Co. (Osan, Korea) and acclimated to the experimental environment for 1 week prior to the experiment. All animals were maintained in an environment at a constant temperature (22 ± 2 °C) and humidity (55% ± 5%).

**Figure 9:**
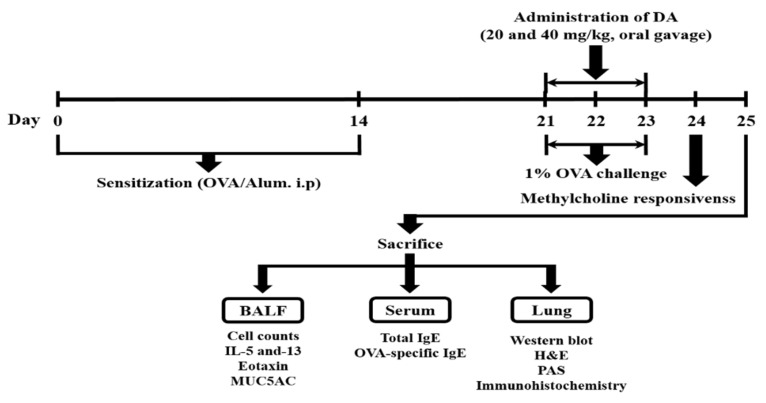
Experimental procedure.

The OVA-induced asthmatic mouse model was generated by a previously described method [35]. We used female mice as an experimental animal, because female mice are more susceptible to the development of allergic airway inflammation than male mice [39,40]. The asthmatic mouse model was sensitized by the intraperitoneal injection of OVA (Sigma-Aldrich, St. Louis, MO, USA, 20 μg) emulsified with aluminum hydroxide (20 mg in PBS, 200 μL) on days 1 and 14, and the sensitized mice inhaled 1% OVA aerosol through a sprayer, twice daily for 30 min on days 21 to 23. The mice were randomly allocated to one of the following (six mice per each group): the normal control group (NC; PBS treatment and PBS inhalation); OVA-induced asthmatic group (PBS treatment and OVA sensitization and inhalation); montelukast treatment group (Mon; 10 mg/kg montelukast treatment, OVA sensitization and inhalation); and D. asperoides C. Y. Cheng et T. M. Ai treatment groups (DA20 and DA40; 20 and 40 mg/kg D. asperoides C. Y. Cheng et T. M. Ai, respectively, OVA sensitization and inhalation). The drug treatments were administered by oral gavage 1 h before the inhalation of 1% OVA aerosol. Montelukast was developed as a cysteinyl leukotriene-1 receptor antagonist and was introduced into the market after successful clinical evaluation in patients with allergic asthma. Twenty-four hours after final OVA challenge, methacholine responsiveness was assessed in conscious and unrestrained mice by means of whole-body plethysmography (OCP3000, Allmedicus, Seoul, Korea). The mouse was placed in a plastic chamber and exposed to methacholine aerosols in increasing concentration (10–30 mg/mL in PBS) for 3 min. After each methacholine challenge, the penh values were measured for 3 min. The experimental procedure is described in Figure 9.

### 4.4. BALF and Serum Analysis

On day 25, bronchoalveolar lavage fluid (BALF) was obtained as previously described [35]. After the mice were anesthetized with alfaxan (Alfaxalone**^®^**, Jurox Pty Ltd., Hunter Valley, Australia), the lungs were lavaged twice with cold PBS (total volume 1.4 mL). BALF was centrifuged at 1500 rpm for 5 min at 4 °C; the supernatant was collected and used for enzyme-linked immunosorbent assay (ELISA) analysis, and the pellets were collected and used for cell analysis. Whole cells from the BALF pellet were attached to a slide glass using a Cytospin (Hanil, Wonju, Korea). BALF was stained with Diff-Quik**^®^** reagent (Sysmax, Kobe, Japan) for differential cell count. The total inflammatory cell counts were evaluated using a cell counting machine (Countess II, Thermo Fisher Scientific, San Diego, CA, USA). The levels of IL-5 (R&D Systems, Minneapolis, MN, USA), IL-13 (R&D Systems), eotaxin (BioSource International, Camarillo, CA, USA), and MUC5AC (MybioSource, San Diego, CA, USA) were measured in BALF supernatant by using an ELISA kit in accordance with the manufacturer’s protocol. Blood collected from the caudal vena cava was centrifuged to obtain serum for analysis. The measurement of IgE level was performed using ELISA kit (BioLegend Inc., San Diego, CA, USA) in accordance with the manufacturer’s protocol. The measurement of OVA-specific IgE was conducted as previously described [41].

### 4.5. Immunoblotting

Lung tissue was collected after BALF sampling and was homogenized using Tissue Lysis/Extraction reagent (Sigma-Aldrich) with a protease inhibitor (Sigma-Aldrich), and the proteins were separated on 8% sodium dodecyl sulfate (SDS)-polyacrylamide gel electrophoresis (PAGE) for 2 h at 100 V and then transferred to a polyvinyl difluoride (PVDF) membranes and incubated with blocking solution (5% skim milk, Millipore Co., Bedford, MA, USA) for 1 h. The membranes were subsequently incubated with specific primary antibodies overnight at 4 °C: anti-p-p65 (1:2000 dilution; Cell Signaling Technologies, Denver, MA, USA), anti-p65 (1:1000 dilution; Cell Signaling Technologies), anti-iNOS (1:1000 dilution; Cell Signaling Technologies), and anti-β-actin (1:2000 dilution; Cell Signaling Technologies). The membranes were incubated with a 1:3000 horseradish peroxidase-conjugated anti-secondary antibody (Thermo Fisher Scientific) for 1 h at room temperature. Finally, each specific band was detected using an enhanced chemiluminescence (ECL) kit (Thermo Fisher Scientific). To determine relative ration of each protein, we measured densitometric band values using ChemiDoc (Bio-Rad Laboratories, Hercules, CA, USA).

### 4.6. Histopathological Analysis

The mouse right lungs were fixed in a 10% neutral buffered formalin and embedded to make in paraffin blocks, which were then sectioned into 4 μm thick slices. The sections were then stained with hematoxylin and eosin (H&E) to evaluate inflammatory responses in lung tissue. In addition, the lung tissues were stained with priodic acid-schiiff (PAS) to detect mucus production. Quantitative analysis of airway inflammation and mucus production was determined using an image analyzer (IMT i-solution software, Vancouver, BC, Cananda).

For immunohistochemical analysis, sections were deparaffinized, dehydrated, and washed in PBS containing 0.05% tween 20 (PBS-T). The slides were incubated to block nonspecific staining for 20 min at room temperature with goat serum. The slides were incubated with primary mouse anti-mouse iNOS antibody (diluted 1:100, Abcam, Cambridge, UK) for 2 h at room temperature. After incubation, they were incubated for 1 h at room temperature with biotinylated secondary antibody, and then incubated with an avidin-biotin-peroxidase complex (Vector Laboratories, Burlingame, CA, USA) for 1 h at room temperature. Then, the slides were washed with PBS-T and incubated with diaminobenzidine (DAB, Abcam) for an additional 5 min.

### 4.7. Statistical Analysis

The data were expressed as the mean ± standard deviation (SD). Statistical analysis was performed using an analysis of variance (ANOVA), followed by a multiple comparison test with Dunnett’s adjustment. A value of *p* < 0.05 indicated a significant difference.
